# LAMTOR/Ragulator regulates lipid metabolism in macrophages and foam cell differentiation

**DOI:** 10.1002/1873-3468.13579

**Published:** 2019-08-26

**Authors:** Giorgia Lamberti, Cedric H. De Smet, Mihaela Angelova, Leopold Kremser, Nicole Taub, Caroline Herrmann, Michael W. Hess, Johannes Rainer, Ivan Tancevski, Rüdiger Schweigreiter, Reinhard Kofler, Thomas Schmiedinger, Ilja Vietor, Zlatko Trajanoski, Christer S. Ejsing, Herbert H. Lindner, Lukas A. Huber, Taras Stasyk

**Affiliations:** ^1^ Division of Cell Biology Biocenter Medical University of Innsbruck Austria; ^2^ Division of Bioinformatics Biocenter Medical University of Innsbruck Austria; ^3^ Division of Clinical Biochemistry Biocenter Medical University of Innsbruck Austria; ^4^ Division of Histology and Embryology Medical University of Innsbruck Austria; ^5^ Division of Molecular Pathophysiology Biocenter Medical University of Innsbruck Austria; ^6^ Department of Internal Medicine Medical University of Innsbruck Austria; ^7^ Division of Neurobiochemistry Medical University of Innsbruck Austria; ^8^ Department of Therapeutic Radiology and Oncology Medical University of Innsbruck Austria; ^9^ Department of Biochemistry and Molecular Biology Villum Center for Bioanalytical Sciences University of Southern Denmark Odense Denmark; ^10^ Austrian Drug Screening Institute, ADSI Innsbruck Austria

**Keywords:** foam cells, LAMTOR, macrophages, organelle proteomics, ragulator, transcriptomics

## Abstract

Late endosomal/lysosomal adaptor and MAPK and mTOR activator (LAMTOR/Ragulator) is a scaffold protein complex that anchors and regulates multiprotein signaling units on late endosomes/lysosomes. To identify LAMTOR‐modulated endolysosomal proteins, primary macrophages were derived from bone marrow of conditional knockout mice carrying a specific deletion of LAMTOR2 in the monocyte/macrophage cell lineage. Affymetrix‐based transcriptomic analysis and quantitative iTRAQ‐based organelle proteomic analysis of endosomes derived from macrophages were performed. Further analyses showed that LAMTOR could be a novel regulator of foam cell differentiation. The lipid droplet formation phenotype observed in macrophages was additionally confirmed in MEFs, where lipidomic analysis identified cholesterol esters as specifically downregulated in LAMTOR2 knockout cells. The data obtained indicate a function of LAMTOR2 in lipid metabolism.

## Graphical abstract

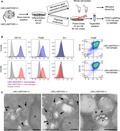

## Abbreviations

**BMDM**, bone marrow‐derived macrophages

**LAMTOR**, Late endosomal/lysosomal Adaptor and MAPK and mTOR activator

**LB**, latex beads

**LE**/**LYS**, late endosomes/lysosomes

**M‐CSF**, macrophage colony‐stimulating factor

Macrophages have a central role in the defense against pathogenic microbes. They display the ability to phagocytose microorganisms and deliver them to the phagolysosome, a specialized organelle responsible for microorganism annihilation. The maturation from phagosome to phagolysosome requires interaction with the endocytic pathway. Endocytic organelles fuse with the maturing phagosome leading to changes in the phagosome proteome composition 1, 2. The close correlation between phagosome maturation and endocytic pathway explains the occurrence of immunodeficiency syndromes caused by defects in proteins governing the fate of endosomes and lysosomes. One of these is the human primary immunodeficiency syndrome caused by a hypomorphic mutation in the endosomal adaptor protein *LAMTOR2* gene 3. The late endosomal/lysosomal adaptor and MAPK and mTOR activator (LAMTOR, also known as Ragulator) is a scaffold protein complex that senses nutrients (e.g., amino acids and lipids) and integrates growth factor signaling 4. The lipid‐modified LAMTOR1 (p18) 5 functions as anchor on late endosomes/lysosomes (LE/LYS) for the remaining LAMTOR subunits, LAMTOR2 (p14) 6, LAMTOR3 (MP1) 7, LAMTOR4 (C7orf59), and LAMTOR5 (HBXIP) 8. LAMTOR was shown to scaffold MEK and ERK on late endosomes 9, 10.LAMTOR interacts also with the Rag GTPases 8, V‐ATPase 11, and SLC38A9 12, 13, 14 and thereby activates lysosomal mTORC1 signaling 15. LAMTOR also regulates late endosomal positioning within the cell, essential for endolysosomal function 15.

We previously described a crucial function of LAMTOR2 for innate and adaptive immunity in patients 3, as well as in specialized immunocompetent cells such as dendritic cells 16, 17, 18 and macrophages 19. In the present study, we investigated the role of LAMTOR2 in the endosomal biogenesis of differentiated macrophages and derived bone marrow‐derived macrophages (BMDM) from established conditional knockout mice (LysMCre) lacking *LAMTOR2* in the monocyte/macrophage lineage 19. Affymetrix‐based transcriptomics analysis was performed on BMDM. LE/LYS were purified using small latex beads (LB) internalization and analyzed by iTRAQ‐based mass spectrometry. Here, we report a novel function of the LAMTOR complex in lipid metabolism, discovered by transcriptomic analyses and organelle proteomics of primary macrophages. Our findings revealed a critical role of the LAMTOR complex in the transdifferentiation of macrophages into foam cells. The lipid droplets formation phenotype was additionally confirmed in mouse embryonic fibroblasts (MEFs). Quantitative lipidome analysis identified cholesterol esters as being specifically downregulated in LAMTOR2KO cells.

## Experimental procedures

### LMC*LAMTOR2* mice

*LAMTOR2f/f*;LysMCre (LMC*LAMTOR2−/−*) mice were obtained as previously described 19. The LysMCre allele was kept hemizygous. Genomic DNA was isolated from ear biopsies with Viagen DirectPCR DNA Extraction System overnight and the genotype was determined by PCR. All animal experiments were approved by the advisory board for animal experimentation of the Medical University of Innsbruck and by the Austrian Ministry for Science and Research (A07/3454).

### Cell isolation and cultivation

Age‐ and sex‐matched LMC*LAMTOR2−/−* and LMC*LAMTOR2+/+* mice were sacrificed by cervical dislocation. Bone marrow cells were recovered from tibiae and femora and erythrocytes were lysed. For differentiation, cells were cultured on non‐tissue culture dishes in RPMI supplemented with 10% FBS, 100 U·mL^−1^ penicillin, 100 U·mL^−1^ streptavidin, and 20 ng·mL^−1^ mouse recombinant macrophage colony‐stimulating factor (M‐CSF) (eBioscience, ​San Diego, CA, USA). After 7 days in culture, cells were grown overnight on tissue culture plates prior to use at a density of 8 × 10^6^ cells/plate. For foam cell formation, 50 μg·mL^−1^ acetylated low‐density lipoprotein was added to the growth medium for 48 h 20.

### Flow cytometry analysis

For FACS analyses, BMDM were washed with PBS, blocked for 10 min with FcyRIII/II‐ block (1 : 500, clone 2.4G2; BD Biosciences, Heidelberg, Germany) and then incubated for 1 h with the specific fluorophore‐conjugated antibodies F4/80‐FITC (Serotec, Puchheim, Germany), CD11b‐PE (eBioscience), Gr1‐APC (BioLegend, Koblenz, Germany), and respective isotype controls. Cells were analyzed by a FacsAria (BD‐Bioscience) and data analysis was performed with flowjo software (Tree Star Inc., Ashland, OR, USA).

### Confocal microscopy

For analysis of foam cell differentiation, BMDM were cultured and fixed for 15 min in 4% paraformaldehyde and lipid droplet staining with BODIPY 493/503 (Invitrogen, Carlsbad, CA, USA) and nuclear staining with Hoechst 3342 (Thermo Scientific, Bremen, Germany) were performed. Confocal stacks were recorded on a Laser Scanning Confocal Microscope (SP5; Leica Microsystems, Wetzlar, Germany) and maximum projections and quantifications were generated using imagej version 1.49a (https://imagej.nih.gov/ij/). For quantification, sum slices projections were made, fluorescence background was subtracted, and integrated density was measured. For detection of lipid droplets in MEFs, cells were fixed and lipid droplets were stained with Oil Red O and pictures were taken with a confocal fluorescence microscope.

### Organelle purification

Internalization of Red‐dyed 80 nm LB (Merck, Darmstadt, Germany) was used to boost endosome formation. LB were internalized for 15 min in macrophages followed by 60 min chase. Endocytic organelles were isolated on a sucrose gradient as previously described 21. The pellets containing purified endosomes were resuspended in homogenization buffer (250 mm sucrose, 3 mm imidazole buffer pH 7.4, 0.5 mm EDTA, 5 mg·mL^−1^ aprotinin, 0.5 mg·mL^−1^ pepstatin, 5 mg·mL^−1^ leupeptin) and protein content was determined with bicinchoninic acid protein assay kit (Pierce, Rockford, lL, USA). Then, 50 μg of protein was loaded on 12% Ready Tris‐HCl gel (Bio‐Rad Laboratories, Feldkirchen, Germany) and run until the dye front penetrated 1 cm into the gel. Coomassie stained areas were excised and in‐gel digested with trypsin, followed by iTRAQ labeling and mass spectrometry.

### Western blot analysis

Whole cell extracts of BMDM, postnuclear supernatant, and LE/LYS samples were separated by SDS/PAGE, blotted and probed with the respective antibodies: LAMTOR2 from Cell Signaling (Frankfurt am Main, Germany), LAMP1 and EEA1 from BD Biosciences, tubulin from Sigma (Vienna, Austria), BiP from Gert Kreibich (New York University School of Medicine), phospho‐p70S6K (pT389), phospho‐S6 (S240/244), and S6 from Cell Signaling, p70S6K and SOAT1 from Santa Cruz (Heidelberg, Germany), MGLL from Thermo Scientific, actin from Merck.

### Electron microscopy

Sapphire coverslips coated with electrospun gelatin fiber meshes were used for culturing BMDM. Cells were incubated with LB for 10–15 min followed by 50–60 min chase, or for 30 min without chase. Sample processing (high‐pressure freezing, freeze substitution, sample rehydration, and immunogold labeling) was described in detail 22. Thawed ultra‐thin cryosections were labeled with rat anti‐LAMP1 (#1D4B, DSHB) 23, visualized with NANOGOLD^®^‐Fab’ goat anti‐rat IgG (H+L) (#2008 Nanoprobes), followed by silver enhancement with HQ‐Silver^®^ (#2012, Nanoprobes) or with rabbit anti‐mouse‐Cathepsin D (courtesy S. Höning) 24 visualized with goat‐anti‐rabbit IgG 5 nm gold (#EM.GAR5; British Biocell, Cardiff, UK).

### Transcriptome analysis

For transcriptomic analysis, BMDM generated from three individual mice of each genotype were pooled, accordingly. Affymetrix‐based gene expression profiles were generated at the Expression Profiling Unit of the Medical University of Innsbruck as described previously 25. In brief, integrity of total RNA was determined by the Bioanalyzer 2100 (Agilent Technologies, Vienna, Austria) and 500 ng of high quality RNA was processed into biotinylated hybridization targets following the manufacturer's protocols and subsequently hybridized onto Affymetrix Mouse Genome 430 2.0 GeneChips. After washing and staining in an Affymetrix fluidic station 450, the microarrays were scanned in an Affymetrix scanner 3000. All further analysis was conducted in R (http://r-project.org) version 3.0.3 using packages from the Bioconductor project 26. Raw microarray data were preprocessed using the GCRMA 27 method. *P*‐values for significance of differential expression were calculated using the moderated *t*‐test 28 and subsequently adjusted for multiple hypothesis testing with the method from Benjamini and Hochberg for a strong control of the FDR 29.

### Mass spectrometry‐based proteomics and data analysis

Protein bands were excised from gel and digested in 50 mm triethylammonium bicarbonate (TEAB) buffer pH 8.5 (1 m solution, Fluka) with trypsin (Promega, Madison, WI, USA) as previously described 30. Extracted peptides were dissolved in 45 μL TEAB buffer and labeled with iTRAQ reagents (AB Sciex) 115 and 116, dissolved in 60 μL absolute ethanol. The samples were incubated at room temperature for 3 h and analyzed using an UltiMate 3000 nano‐HPLC system coupled to a Q Exactive Plus mass spectrometer (Thermo Scientific) equipped with a Nanospray Flex ionization source. The peptides were separated on a homemade fritless fused silica microcapillary column (75 μm i.d. ×280 μm o.d. ×10 cm length) packed with 3 μm C18 material (Reprosil, Dr. Maisch HPLC, Ammerbuch‐Entringen, Germany). Solvents for HPLC were 0.1% formic acid (solvent A) and 0.1% formic acid in 85% acetonitrile (solvent B). The gradient profile was as follows: 0–2 min, 4% B; 2–55 min, 4–50% B (or 2–85 min, 4–50% B); 55–60 min, 50–100% B, and 60–65 min, 100% B. The flow rate was 250 nL·min^−1^. The mass spectrometer was operating in the data‐dependent mode selecting the top 12 most abundant isotope patterns with charge > 1 from the survey scan with an isolation window of 1.2 *m/z* ratio. Survey full scan MS spectra were acquired from 300 to 1750 *m/z* at a resolution of 70 000 (60 000) with a maximum injection time (IT) of 120 ms, and automatic gain control (AGC) target 1e6. The selected isotope patterns were fragmented by HCD with normalized collision energy of 25 at a resolution of 17 500 (15 000) with a maximum IT of 60 ms, and AGC target 5e5.

iTRAQ data analysis was performed using MaxQuant v1.5.2.6 31. The UniProt protein annotation for *Mus musculus* (last update Jan 2014, http://uniprot.org), accounting to 44 455 protein sequences, was used for peptide identification. Precursor and fragment mass tolerance were set to 10 p.p.m. and 0.02 Da, respectively, and up to two missed cleavages were allowed. Carbamidomethylation of cysteine was selected as fixed modification, oxidation of methionine and iTRAQ4plex115 and iTRAQ4plex116 labeling at the N terminus, lysine and tyrosine residues were set as variable modifications. Peptide identifications were filtered at 1% FDR. All samples were analyzed together in one run, in order to ensure that the same peptides were consistently assigned to the same proteins. The biological replicates were annotated as separate experiments. The technical replicates were analyzed together, with the argument ‘match from and to’, in order to increase the power of peptide detection and quantification. All other settings were default. In order to ensure sufficient reliability for all samples, quality control was performed by comparing identified MS/MS scans, protein digestion, missed cleavages, and label incorporation, and correlation of protein ratios between different replicates. We performed two independent iTRAQ‐based MS experiments to analyze samples representing two biological replicates of pooled organelles isolated from BMDM derived from three individual mice of each genotype (LMCLAMTOR2−/− and LMCLAMTOR2+/+, six mice in total for each genotype). Reproducible protein regulation was detected using the following filtering criteria: each protein had at least one unique peptide, iTRAQ‐quantified in two biological replicates, and a cutoff was limited to log_2_ > 0.5 of differential protein expression in two experiments.

### GO enrichment analysis

The Gene Ontology (GO) enrichment analysis was performed on differentially regulated genes and proteins in GO Biological Processes, Cellular Components, and Molecular Function 32. Additionally, KEGG pathway analysis was performed on the list of regulated proteins 33. All adjusted *P*‐values below FDR 0.05 were reported.

### Lipid analysis by thin‐layer chromatography

Bone marrow‐derived macrophages were cultured and treated as described above. Cell pellets were resuspended in 300 μL PBS, and 1 mL chloroform:methanol:acetate 10 : 20 : 0.5 was added; tubes were vortexed and kept on ice for 10 min. One milliliter chloroform and 1 mL water were added subsequently, each followed by vortexing. Tubes were centrifuged for 1 min at 210 ***g***. The hydrophobic organic phase and water phase were collected and were dried in a SpeedVac Concentrator (Thermo Scientific). The water phase samples were used to determine protein concentration by bicinchoninic acid Protein Assay Kit (Pierce) for normalization. Dry lipid extracts were dissolved in 50 μL chloroform, and the amount of extract corresponding to 1 mg protein was loaded on a TLC silica gel plate (Merck) to separate neutral lipids using petroleum ether : ethyl methyl ketone : acetate 95 : 10 : 1 as running buffer. The front was allowed to run up to 1 cm below the top of the plate, the plate was dried and stained with 3% phosphomolybdate (Sigma‐Aldrich) for 60 s, and developed for 5 min at 120 °C. All solvents used were HPLC grade and obtained from Sigma‐Aldrich.

### Mass spectrometry‐based lipidomics

LAMTOR2−/− MEFs, control LAMTOR2f/‐ MEFs 9, and LAMTOR2−/− MEFs reconstituted by expression of LAMTOR2‐GFP 34 were grown to subconfluency, and lipids were extracted and analyzed by shotgun lipidomics as previously described 35. In total, two biological and two technical replicates per genotype were quantified and analyzed by applying an unpaired Student's *t*‐test.

## Results and Discussion

### Transcriptome analysis of LMC*LAMTOR*2−/− and LMC*LAMTOR2*+/+ BMDM

**Figure 1:**
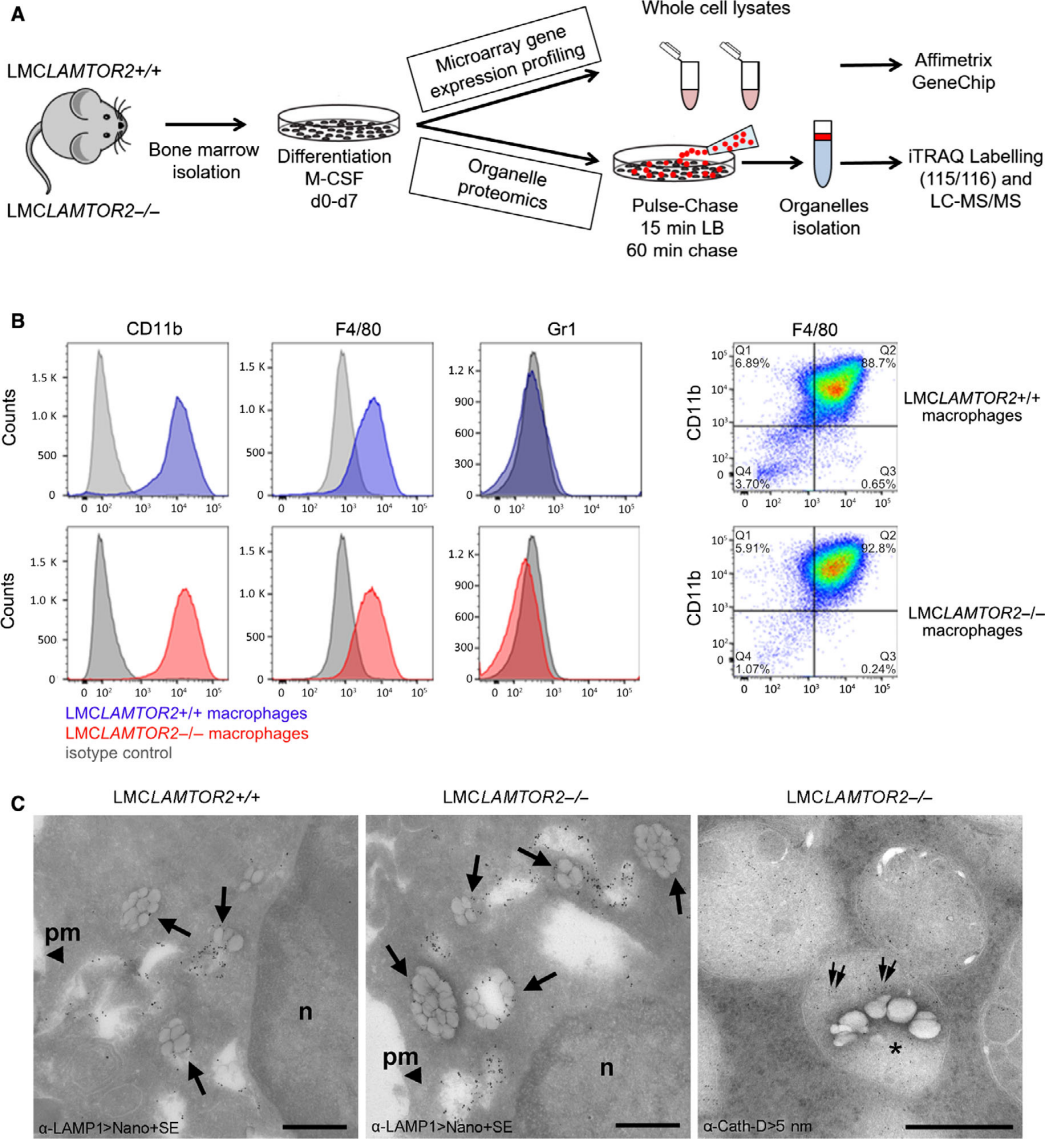
Experimental design. (A) Schematic illustration of transcriptomics and endosome proteomics experimental setup. Hematopoietic stem cells from LAMTOR2‐deficient (LMC*LAMTOR2*−*/*−) and control (LMCLAMTOR2+/+) mice were extracted and differentiated *in vitro* with recombinant M‐CSF for 7 days. For transcriptomic analysis, macrophages whole cells lysates were prepared, RNA was extracted and hybridized onto an Affymetrix GeneChip, and microarrays gene expression profiling was performed. For LE proteomics, BMDM were fed with LB for 15 min followed by 60 min chase without LB and LE/LYS were separated on density gradients. Differentially expressed proteins were quantified by iTRAQ labeling and LC‐MS/MS. (B) Surface expression of F4/80, CD11b, and Gr1. BMDM from LMC*LAMTOR2*+/+ and LMC*LAMTOR2*−*/*− mice were stained for F4/80, CD11b, and Gr1 and the surface expression was determined as shown in the histogram (blue: LMC*LAMTOR2*+/+, red: LMC*LAMTOR2*−*/*−, gray: isotype control). Right panels: dot plot showing macrophages double positive for CD11b and F4/80. (C) Immunoelectron microscopic analysis of macrophages with internalized LB. LB within LAMP1‐positive LE/LYS compartments (arrows) as seen in LMCLAMTOR2+/+ and LMCLAMTOR2−/− BMDM, incubated for 15 min with LB and chased for 60 min; n, nucleus; pm, plasma membrane; visualization of primary antibodies with Nanogold‐conjugates plus silver enhancement (Nano+SE); scale bars, 500 nm. Right panel, LB within a LE compartment (asterisk) with distinct Cathepsin D immunolabeling (double arrows; visualization with 5 nm gold conjugates); LMCLAMTOR2−/− BMDM incubated with LB for 30 min without chase; scale bar, 200 nm.

To identify LAMTOR‐modulated cellular transcripts and potential downstream targets, Affymetrix‐based transcriptomics analysis was performed. In addition, and complementary to the transcriptome analysis, quantitative iTRAQ‐based organelle proteomics analysis of endolysosomes derived from LAMTOR2‐deficient macrophages has been performed (Fig. 1A). BMDM were obtained from LMC*LAMTOR2*−*/*− and LMC*LAMTOR2+/+* mice and differentiated in the presence of M‐CSF 36. Differentiation efficiency was tested by flow cytometry analysis using the mature macrophage markers F4/80 and CD11b and the negative control Gr1 (Fig. 1B). Macrophages obtained from both mice lines were comparable in terms of surface marker expression and resulted as efficiently differentiated after 7 days in culture.

To understand the overall transcriptional response in LAMTOR2‐depleted macrophages, Affymetrix GeneChip analysis was performed. The gene expression profiles of LMC*LAMTOR2*+/+ and LMC*LAMTOR2*−/− BMDM revealed 178 differentially expressed genes (|M| ≥ 1 and BHp ≤ 0.01, representing a ≥ 2‐fold change in expression and a ≤ 1% false discovery rate, respectively), 127 of which were upregulated, and 51 genes were downregulated in LAMTOR2−/− cells (Table S1). Surprisingly, on the transcriptome levels in LAMTOR2−/− BMDM, the number of regulated genes involved in cellular lipid metabolic processes was very pronounced. Upregulated lipid metabolism genes included *ABCG1,* that encodes the ATP‐binding cassette subfamily G member 1, a transporter involved in macrophage lipid homeostasis; *FABP4*, fatty acid‐binding protein 4; and *ApoE*, apolipoprotein that mediates the binding and catabolism of lipoprotein particles. GO analysis of differentially expressed genes identified a specific enrichment of terms related to lipid metabolism, such as sterol esterification, foam cell differentiation, HDL and LDL particle remodeling, positive regulation of cholesterol efflux and reverse cholesterol transport, and negative regulation of lipid storage (Table S2).

### Proteome analysis of late endosomes from LMC*LAMTOR2*−*/*− and LMC*LAMTOR2*+/+ BMDM

To complement the transcriptomics data on the global cellular response, we investigated the effect of LAMTOR2 deletion on the LE/LYS proteome of murine macrophages. We developed a method to isolate endocytic organelles from primary murine macrophages optimizing the previously described method based on LB fed cultured cells 21, 37 and combined it with an iTRAQ‐based quantitative proteomic approach (Fig. 1A). BMDM were fed with 80 nm LB for 15 min followed by 60 min chase. Efficient uptake and intracellular trafficking of the beads were demonstrated by immunoelectron microscopy, where at the chosen time point, numerous LB localized within LE/LYS compartments, of which a large proportion was clearly positive for the late endosomal marker LAMP1 in both genotypes (Fig. 1C). The LE/LYS nature of these latex‐harboring organelles was further supported by Cathepsin D immunoelectron microscopy (Fig. 1C, right panel). LB‐containing endosomes were isolated on sucrose gradients as previously described 21, 37. The enrichment of LE/LYS and the absence of major contaminants in our preparations were confirmed by western blot analyses with antibodies against different cellular compartments (Fig. S1).

The proteins obtained by the LE/LYS preparation were separated by SDS/PAGE, digested, iTRAQ labeled, and analyzed by LC‐MS/MS. This approach led to the identification and quantification of 1101 proteins, among which all five subunits of the LAMTOR complex were present (Table S3). Small LB internalization led to a decrease in the buoyant density of LE/LYS that allowed separation by floatation in sucrose gradients from contaminants of otherwise similar density 21, 37. High enrichment of LE/LYS achieved in a single purification step provided significant advantages compared to the conventional protocols. The number of LE/LYS proteins identified and quantified here (1101) was comparable with 1565 proteins identified in the lysosomal proteome upon complex fractionation and subsequent 36 MS analyses 38 as well as with a study identifying 2385 lysosomal proteins by a combination of subcellular and biochemical fractionations followed by 959 MS analyses 39. An in‐depth interpretation of the revealed organelle proteome, in terms of specificity of protein enrichment, would require further validation that lies beyond the scope of this work.

As a quality control, we compared the 1101 quantified proteins with the endosomal proteome of J744 macrophages described by Duclos *et al*. 37. We found that 195 proteins out of 265 (74%) identified by Duclos *et al*. matched our iTRAQ results (Table S4). Furthermore, 145 proteins of the 191 (76%) assigned by Duclos *et al*. 37 to the late endosome overlapped with our late endosomal proteome (Table S4), confirming the high quality of our preparation. Of 1101 proteins quantified with our iTRAQ approach, 31 proteins were found to be differentially regulated in the LMC*LAMTOR2*−/− sample compared to the LMC*LAMTOR2*+/+ control (Table S3).

To understand the biological roles of the differentially expressed proteins in LMC*LAMTOR2*−/− LE/LYS, we conducted GO enrichment analyses (Table S3). As expected, mTOR signaling was among the most enriched regulated GO terms. The depletion of the remaining components of the LAMTOR complex from endosomes was consistent with our previous observations 34, 40. Interestingly, in addition to mTOR signaling and the protein complex scaffolding function, regulation of lipid metabolism was specifically enriched. Cholesterol binding, lipid binding, and phosphatidylserine binding were among the Molecular Functions regulated by LAMTOR2 depletion in endosomes (Table S3). Furthermore, cholesterol metabolism and sphingolipid metabolism were among the regulated KEGG pathways.

### LAMTOR2 regulates downstream mTORC1 signaling and key enzymes of lipid metabolism

**Figure 2:**
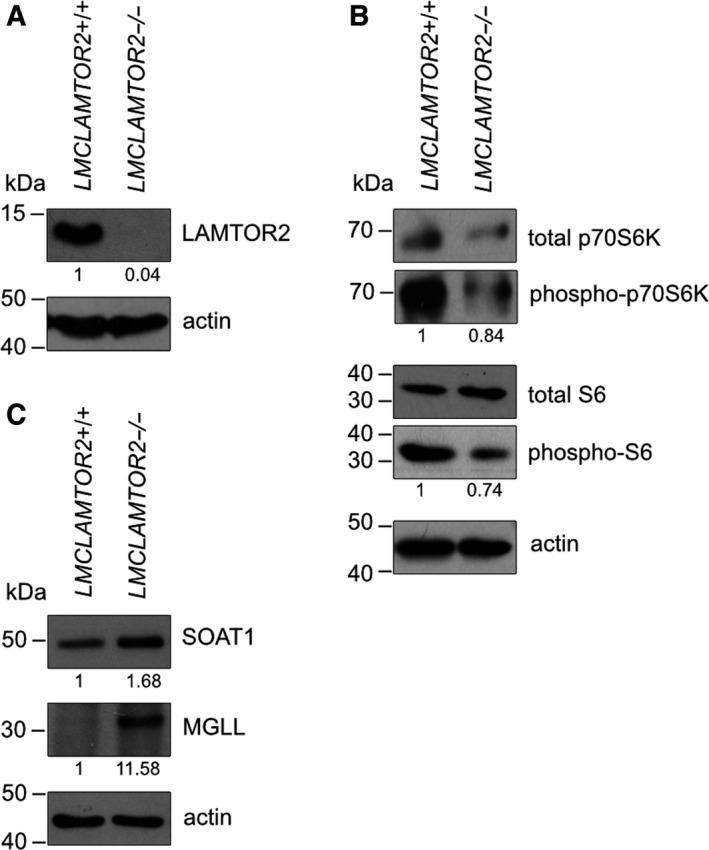
LAMTOR2 regulates downstream mTORC1 signaling and key enzymes of lipid metabolism. (A) Knockout efficiency of LAMTOR2 in macrophages derived from LMC*LAMTOR2*+/+ and LMC*LAMTOR2*−/− mice. (B) Western blot analyses of mTORC1 targets—phospho‐p70S6K and downstream phospho‐S6. (C) Western blotting of SOAT1 and MGLL. Whole BMDM cell lysates were analyzed and actin was used as loading control. Relative values (shown as fold change, *n* = 3) were normalized to actin as loading control and to total p70S6K and total S6 for corresponding phosphorylated proteins.

Consistent with the well‐established function of the LAMTOR/Ragulator complex as a scaffold for LE/LYS mTOR signaling, phosphorylation of a direct mTORC1 target p70S6 kinase as well as the downstream ribosomal protein S6 were significantly decreased in LAMTOR2−/− macrophages, as revealed by western blot analyses (Fig. 2A,B). More specifically, we aimed to confirm targets involved in lipid metabolism as predicted by transcriptomics and organelle proteomics. Upregulated lipid metabolism‐related proteins in the LE/LYS proteome of LAMTOR2−/− macrophages included sterol O‐acyltransferase‐1 SOAT1/ACAT‐1 and monoacylglycerol lipase MGLL. Hence, these two key enzymes were detected as regulated also on transcriptional levels and were therefore selected for further analyses. On LE/LYS protein levels, SOAT1 was upregulated; MGLL was detected and quantified as upregulated only in one biological replicate (Table S3). Importantly, the upregulation of both proteins in LAMTOR2−/− macrophages was confirmed by western blotting analysis (Fig. 2C). Interestingly, these proteins are directly connected to lipid metabolism, namely SOAT1 catalyzing the formation of cholesterol esters by transfer of acyl chains to cholesterol, whereas MGLL being involved in the triacylglycerol degradation pathway. SOAT1 and MGLL have been implicated in regulation of foam cell differentiation and atherosclerosis 41, 42. SOAT1 activity has also been shown to deter atherosclerosis, by regulating cholesterol efflux from macrophages 42.

### LAMTOR2 plays a role in foam cell differentiation

**Figure 3:**
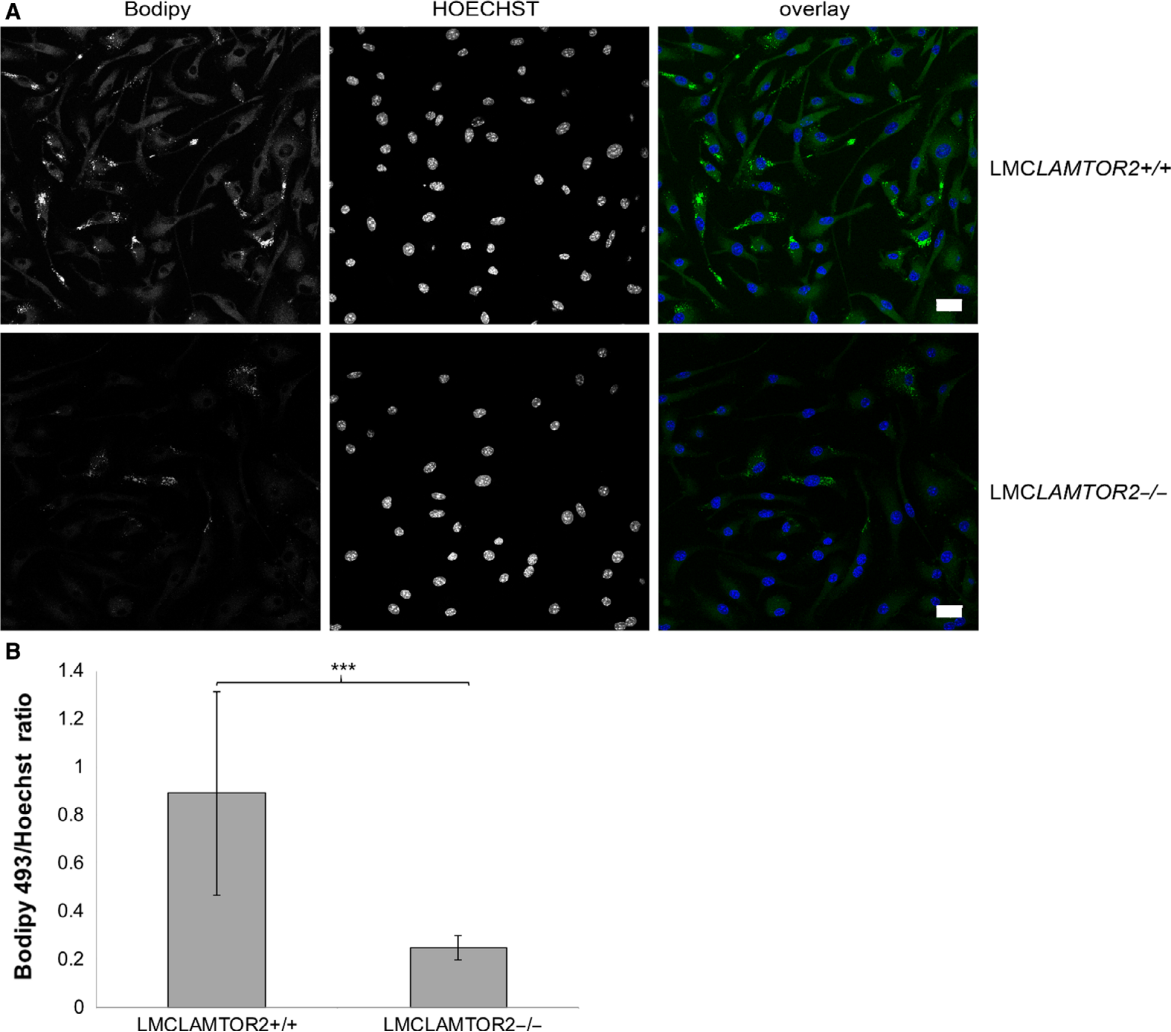
LAMTOR2 regulates foam cell differentiation. (A) Confocal microscopy analysis of LMC*LAMTOR2*+/+ and LMC*LAMTOR2*−*/*− foam cells. Cells were fixed and stained with BODIPY 493/503 and Hoechst. Scale bar, 20 μm. (B) Relative fluorescence intensity quantification expressed as ratio of integrated density of BODIPY 493/503 to Hoechst fluorescent signal (*n* = 10 images containing at least 100 cells for each cell type, *P* = 0.0009).

To confirm and substantiate our findings on a possible role of LAMTOR in lipid metabolism, we decided to analyze foam cell differentiation as it was also one of the significantly enriched biological pathways identified with Affymetrix GeneChip Analysis (Table S2). In this experiment, we visualized lipid droplets in BMDM by fluorescence staining with BODIPY 493/503 (Fig. 3A). Lipid droplets are organelles that store neutral lipids, including triglycerides and cholesterol esters. Quantification of the fluorescent signal showed fourfold less efficient accumulation of lipids in LAMTOR2−/− macrophage‐derived foam cells in comparison to the control cells (Fig. 3B). These experiments clearly confirmed the bioinformatically predicted LAMTOR2 functions in lipid metabolism and regulation of macrophage‐derived foam cell differentiation, suggesting that LAMTOR regulates cholesterol homeostasis at the LE/LYS membrane, consistent with the recent discovery of LAMTOR‐interacting SLC38A9 protein as a cholesterol sensor 43.

**Figure 4:**
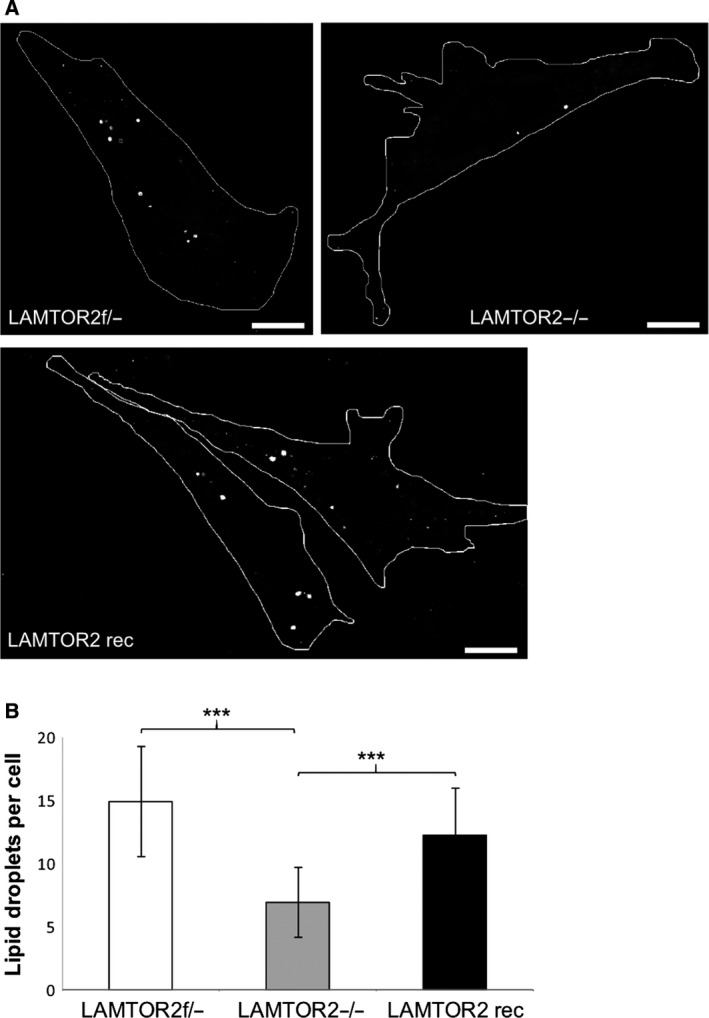
Lipid droplets are reduced in LAMTOR2−/− MEFs. (A) LAMTOR2f/−, LAMTOR2−/−, and LAMTOR2 reconstituted by expression of LAMTOR2‐GFP MEFs were fixed, lipid droplets were stained with Oil Red O and pictures were taken with a confocal fluorescence microscope. (B) Lipid droplet count per cell of the indicated cell types. Student's *t*‐test *P* < 0.001, ***.

Bone marrow‐derived primary macrophages have technical limitations for re‐expression of LAMTOR2. In order to address the specificity of the observed phenotype, we made use of previously established mouse embryonic fibroblasts (MEFs) bearing either a single WT allele (f/‐), or complete LAMTOR2 deletion (−/−) 9 and a rescued cell line established by re‐expressing pEGFP‐LAMTOR2 in LAMTOR2−/− MEFs 34. The lipid droplet formation phenotype described in primary macrophages was reproducibly detected also in MEFs, as demonstrated in Fig. 4A. A significant, more than twofold, reduction in the amount of Oil Red O‐positive lipid droplets was observed in LAMTOR2−/− MEFs compared to the LAMTOR2f/‐ control MEFs. Most importantly, the amount of lipid droplets in LAMTOR2 reconstituted cells was comparable to the LAMTOR2f/‐ control MEFs, as quantified in Fig. 4B.

**Figure 5:**
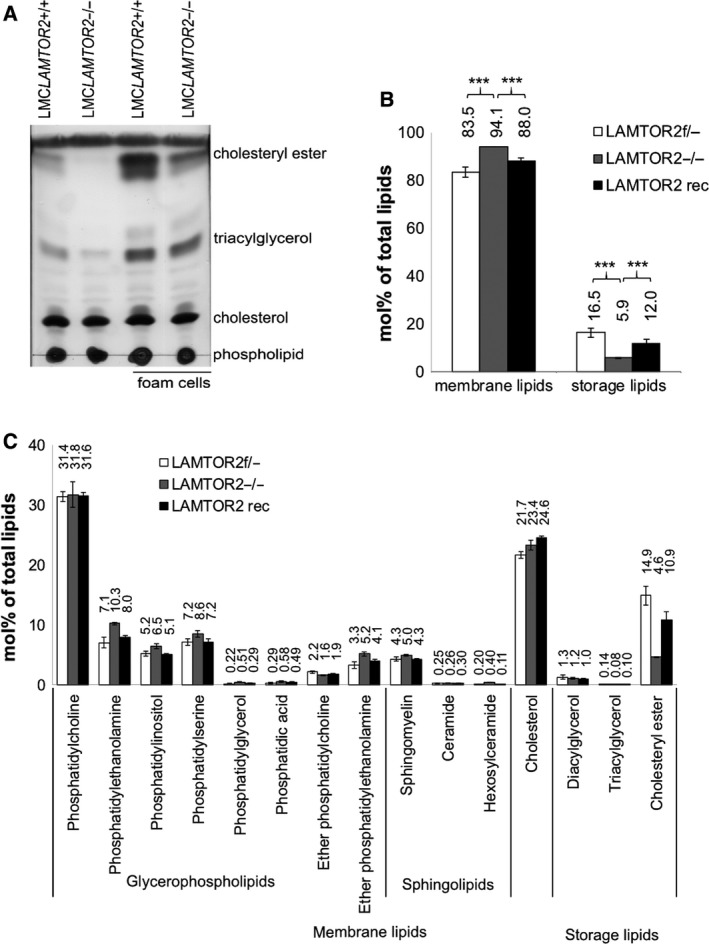
LAMTOR2 regulates cellular lipid composition. (A) Thin‐layer chromatography of whole cell lysates from LMC*LAMTOR2*+/+ and LMC*LAMTOR2*−*/*− BMDM. Foam cells were cultured in the presence of 50 μg·mL^−1^ acetylated LDL. (B) Molar abundance (%) of membrane lipids and storage lipids in LAMTOR2−/− MEFs versus control LAMTOR2f/− MEFs and LAMTOR2−/− MEFs reconstituted by expression of LAMTOR2‐GFP, analyzed by shotgun lipidomics. Student's *t*‐test *P* < 0.001, ***. (C) More detailed view zooming in on lipid classes.

These results were additionally corroborated by thin‐layer chromatography analysis of cellular lipid extracts of BMDM treated with acetylated LDL for 48 h. Both untreated as well as differentiated foam cells derived from LAMTOR2−/− BMDM displayed a reduced storage lipid content (cholesterol ester and triacylglycerol) when compared to LAMTOR2+/+ control cells (Fig. 5A).

In order to unequivocally identify the lipid species regulated in LAMTOR2 KO cells, quantitative mass spectrometry‐based lipidomics was performed on MEFs (LAMTOR2f/‐ as control, LAMTOR2−/− and reconstituted LAMTOR2 cells). Deletion of LAMTOR2 resulted in a 50% decrease in the abundance of storage lipids (i.e., the lipid fraction corresponding to cholesteryl ester, diacylglycerol, and triacylglycerol), and in a 10% increase in the membrane lipid content (i.e., the lipid fraction corresponding to glycerophospholipids, sphingolipids, and cholesterol) (Fig. 5B). Detailed lipidomics analyses revealed a prominent reduction in neutral lipid cholesteryl esters, in LAMTOR2−/− MEFs compared to LAMTOR2f/‐ control, whereas total free cholesterol content was unchanged (Fig. 5C). Furthermore, upregulation of different membrane lipid classes, namely phosphatidylethanolamine and ether phosphatidylethanolamine, phosphatidylinositol, phosphatidylglycerol, and hexosylceramide, was observed in LAMTOR2−/− MEFs (Fig. 5C, unpaired Student's *t*‐test *P* < 0.01). Importantly, the specific regulation of lipids in LAMTOR2−/− was rescued in reconstituted MEFs (Fig. 5B,C). Mechanistically, our results indicated that upregulation of SOAT1 and MGLL might contribute to the lipid phenotype in LAMTOR2‐deficient BMDM. Interestingly, previously established SOAT1 KO macrophages were rich in intracellular vesicles, which may serve to sequester cholesterol into a pool that is unavailable for cholesterol efflux 42. We hypothesize that the upregulation of SOAT1 is a way for the cells to compensate the storage lipid accumulation caused by the KO of LAMTOR2. Whether or not these or any other regulated proteins/transcripts contribute to the detected phenotypes requires further investigations.

The observed phenotypes in macrophage derived foam cell differentiation suggested that LAMTOR might play a role in the activation of BMDM. Of note, on the whole organism level, macrophages are generated by the differentiation of bone marrow‐derived monocytes when these enter the peripheral tissues. Foam cells are defined as the fat‐laden differentiated macrophages and serve as the hallmark of early stage atherosclerotic lesions 44, 45, 46. Taken together, our results showed an important and so far unknown role for the LAMTOR complex in foam cell formation suggesting that it might regulate cellular lipid metabolism. Therefore, our findings propose the LAMTOR complex as a potential therapeutic target for modulating macrophage function in atherosclerosis and other lipid storage disorders.

## Author contributions

GL, CHDS, and LAH designed the experiments. GL, CHDS, LK, NT, CH, MWH, RS, and TSc performed the experiments. GL, CHDS, MA, JR, RK, ZT, CSE, HHL, and TSt analyzed data. GL, CHDS, LAH, and TSt wrote the manuscript.

## Supporting information


**Fig. S1.** Western blot analysis of PNS and endosomes preparations.

**Table S1.** Complete results of Affymetrix gene chip analysis.

**Table S2.** List of GO terms regulated in the differentially expressed genes in LMCLAMTOR2−/− BMDM.

**Table S3.** List of proteins quantified with iTRAQ.

**Table S4.** Comparison of late endosomal proteins quantified with iTRAQ in this study and the endosomal proteome quantified by Duclos *et al*.

## Data Availability

The mass spectrometry proteomics data have been deposited to the ProteomeXchange Consortium (http://www.proteomexchange.org) via the PRIDE 47 partner repository with the dataset identifier PXD009148.
