# Supplementary figures and images for: LAMTOR/Ragulator regulates lipid metabolism in macrophages and foam cell differentiation
Source: FEBS Lett. 2019 Aug 26;594(1):31–42. doi: 10.1002/1873-3468.13579 (PMC7003824; doi:10.1002/1873-3468.13579)

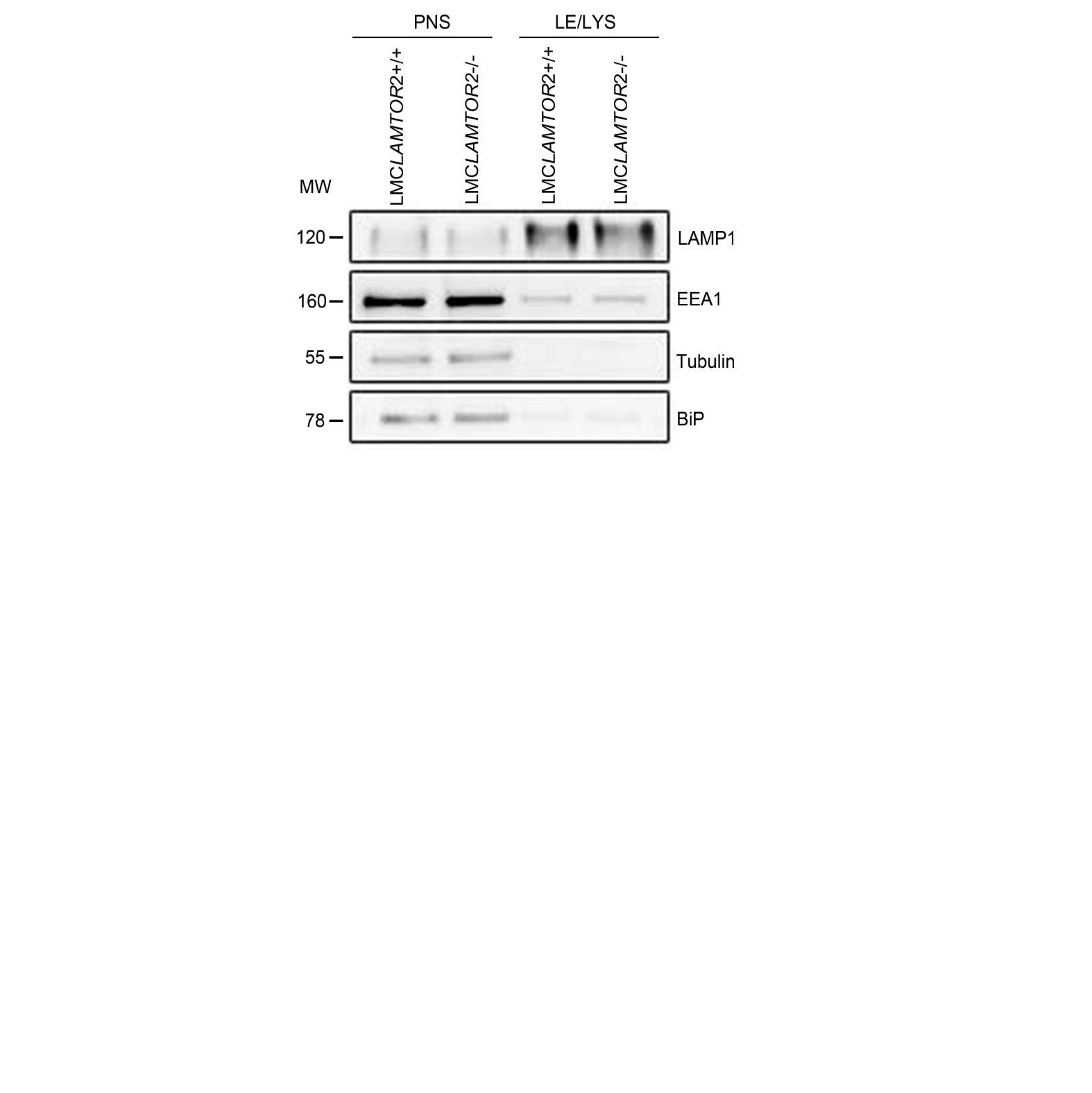

Supplement: Supplementary file 1 — Fig. S1. Western blot analysis of PNS and endosomes preparations. [file FEB2-594-31-s001.tiff]
