# Supplementary material for: LAMTOR/Ragulator regulates lipid metabolism in macrophages and foam cell differentiation
Source: FEBS Lett. 2019 Aug 26;594(1):31–42. doi: 10.1002/1873-3468.13579 (PMC7003824; doi:10.1002/1873-3468.13579)
Supplement: Supplementary file 6 [file FEB2-594-31-s002.docx]

**Supplemental Figure S1. Western blot analysis of PNS and endosomes preparations.**  LAMP1 (late endosomes) was enriched in the LE/LYS fractions, while EEA1 (early endosomes), tubulin (cytoskeleton) and BiP (ER) were depleted.

**Supplemental Table S1.** Complete results of Affymetrix gene chip analysis.

**Supplemental Table S2.** List of GO terms regulated in the differentially expressed genes in LMCLAMTOR2-/- BMDM.

**Supplemental Table S3.** List of proteins quantified with iTRAQ. List of differentially expressed proteins. List of GO terms regulated in the differentially expressed LMCLAMTOR2-/- late endosomal proteins.

**Supplemental Table S4.** Comparison of late endosomal proteins quantified with iTRAQ in this study and the endosomal proteome quantified by Duclos et al.
